# Development of cross-resistance by *Aspergillus fumigatus* to clinical azoles following exposure to prochloraz, an agricultural azole

**DOI:** 10.1186/1471-2180-14-155

**Published:** 2014-06-11

**Authors:** Isabel Faria-Ramos, Sofia Farinha, João Neves-Maia, Pedro Ribeiro Tavares, Isabel M Miranda, Letícia M Estevinho, Cidália Pina-Vaz, Acácio G Rodrigues

**Affiliations:** 1 Microbiology Department, Faculty of Medicine, University of Porto, 4200-319 Porto, Portugal; 2 Cardiovascular Research & Development Unit, Faculty of Medicine, University of Porto, Porto, Portugal; 3 CINTESIS - Center for Health Technology and Services Research, Faculty of Medicine of the University of Porto, Porto, Portugal; 4 Department of Biology and Biotechnology, CIMO-Mountain Research Center, Agricultural College of Bragança, Polytechnic Institute of Bragança, Porto, Portugal; 5 Microbiology Department, Centro Hospitalar de São João, Porto, Portugal; 6 Burn Unit, Centro Hospitalar de São João, Porto, Portugal

**Keywords:** Aspergillus fumigatus, Cross-resistance, Clinical and agricultural azoles

## Abstract

**Background:**

The purpose of this study was to unveil whether azole antifungals used in agriculture, similar to the clinical azoles used in humans, can evoke resistance among relevant human pathogens like *Aspergillus fumigatus,* an ubiquitous agent in nature. Additionally, cross-resistance with clinical azoles was investigated. Antifungal susceptibility testing of environmental and clinical isolates of *A. fumigatus* was performed according to the CLSI M38-A2 protocol. *In vitro* induction assays were conducted involving daily incubation of susceptible *A. fumigatus* isolates, at 35°C and 180 rpm, in fresh GYEP broth medium supplemented with Prochloraz (PCZ), a potent agricultural antifungal, for a period of 30 days. Minimal inhibitory concentrations (MIC) of PCZ and clinical azoles were monitored every ten days. In order to assess the stability of the developed MIC, the strains were afterwards sub-cultured for an additional 30 days in the absence of antifungal. Along the *in vitro* induction process, microscopic and macroscopic cultural observations were registered.

**Results:**

MIC of PCZ increased 256 times after the initial exposure; cross-resistance to all tested clinical azoles was observed. The new MIC value of agricultural and of clinical azoles maintained stable in the absence of the selective PCZ pressure. PCZ exposure was also associated to morphological colony changes: macroscopically the colonies became mostly white, losing the typical pigmentation; microscopic examination revealed the absence of conidiation.

**Conclusions:**

PCZ exposure induced *Aspergillus fumigatus* morphological changes and an evident increase of MIC value to PCZ as well as the development of cross-resistance with posaconazole, itraconazole and voriconazole.

## Background

The ubiquitous saprophytic mould *Aspergillus fumigatus* is known to cause a spectrum of diseases in humans, including allergic syndromes, noninvasive infections, and invasive aspergillosis, a condition associated with significant morbidity and mortality [1]_._*A. fumigatus* is one of the human pathogenic fungi that have a natural habitat in the environment, including soil and plants [2]. Some members of the azole drug class, which includes voriconazole (VRC) and posaconazole (POS), have been shown to be effective in the treatment of invasive aspergillosis [3], and for a long time, azole resistance among clinical *A. fumigatus* isolates was considered to be an uncommon finding. However, multiazole resistance is emerging and is increasingly recognized as a cause of treatment failure [4,5]. In agriculture, thousands of tons of azoles are sold annually for the purpose of plant protection, either to prevent or to control fungal growth that can cause extensive loss of crops or to ease the problem of postharvest spoilage of plants and fruits [6]. The mechanism of action of all azoles - irrespectively of their chemical structure and variable biological properties - is based on its interference with the activity of fungal lanosterol 14 alpha-demethylase, an enzyme encoded by *Cyp51A* gene in *A. fumigatus* that is responsible for the transformation of lanosterol in ergosterol, an essential component of the fungal cytoplasmatic membrane. The inhibition of ergosterol formation results in cell membrane disorganization and impairment of fungal growth. Therefore, azoles are considered fungistatic rather than fungicidal, and it is well known that a strong and persistent antimicrobial pressure can lead to the selection of resistant clones, particularly if the drug effect is static rather than microbicidal [7]. Since azoles are the mainstay treatment for both human and agricultural fungal diseases, a major concern is the predictable emergence of cross-resistance to clinical *A. fumigatus* isolates that is already observed in several countries, driven by the massive use of azole fungicides in agriculture, which have the same mechanism of action as those used in humans [7-11]. The aim of our study was to investigate whether Prochloraz (PCZ), an azole extensively used in agriculture, could be associated with the development of cross-resistance to clinical azoles among *A. fumigatus*.

## Results and discussion

The three isolates developed a progressive increment of PCZ minimal inhibitory concentrations (MIC) value. In addition, a concomitant increase of the MIC of VRC, POS and Itraconazole (ITZ) was also observed (Table 1). During the induction assay, MIC of PCZ increased 256 times from day 0 until day 30. Concerning the clinical azoles, cross-resistance was developed since all isolates changed from a susceptible to a resistant phenotype, according to Meletiadis and colleagues [12].

**Table 1:** **Susceptibility pattern of tested *A. fumigatus *** **isolates to Prochloraz and clinical azoles**

***A. fumigatus *** **isolate**	**Time of exposure (days)**					
		**MIC (mg/L)**				
		**PCZ**	**VRC**	**POS**	**ITZ**	**FLC**
**LMF05**	**0**	0.125	0.125	0.25	2	>64
	**10**	0.25	0.25	0.5	2	>64
	**20**	8	2	1	4	>64
	**30**	32	8	2	8	>64
	Ø**30**	32	2	2	2	>64
**LMF11**	**0**	0.125	0.25	0.125	0.5	>64
	**10**	0.125	2	0.25	1	>64
	**20**	8	8	1	2	>64
	**30**	32	>16	4	4	>64
	Ø**30**	32	2	1	0.5	>64
**LMN60**	**0**	0.25	0.25	0.125	0.25	>64
	**10**	4	8	0.25	1	>64
	**20**	8	8	0.5	2	>64
	**30**	64	>16	4	4	>64
	Ø**30**	64	2	1	0.25	>64

PCZ, Prochloraz; VRC, Voriconazole; POS, Posaconazole; ITZ, Itraconazole; FLC, Fluconazole; Ø, MIC after 30 days of culture in the absence of PCZ.

**Figure 1:**
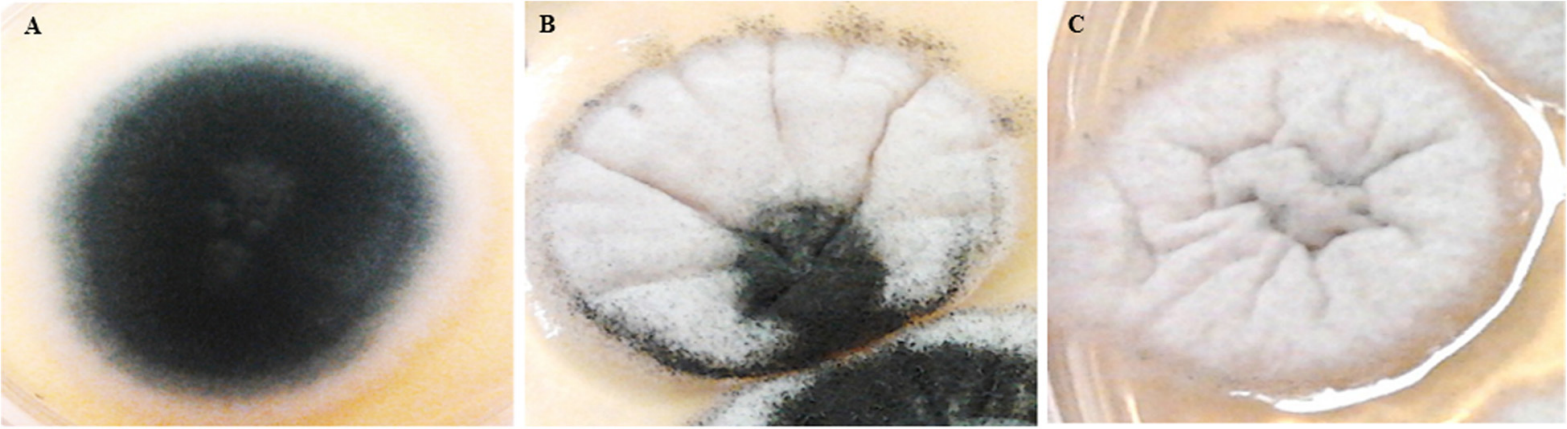
**Photographs of Sabouraud dextrose agar plates showing macroscopic morphological changes of colonies of *A. fumigatus *following exposure to subinhibitory concentration of PCZ. A**. Initial morphological aspect (control). **B**. After fifteen days. **C**. After thirty days.

**Figure 2:**
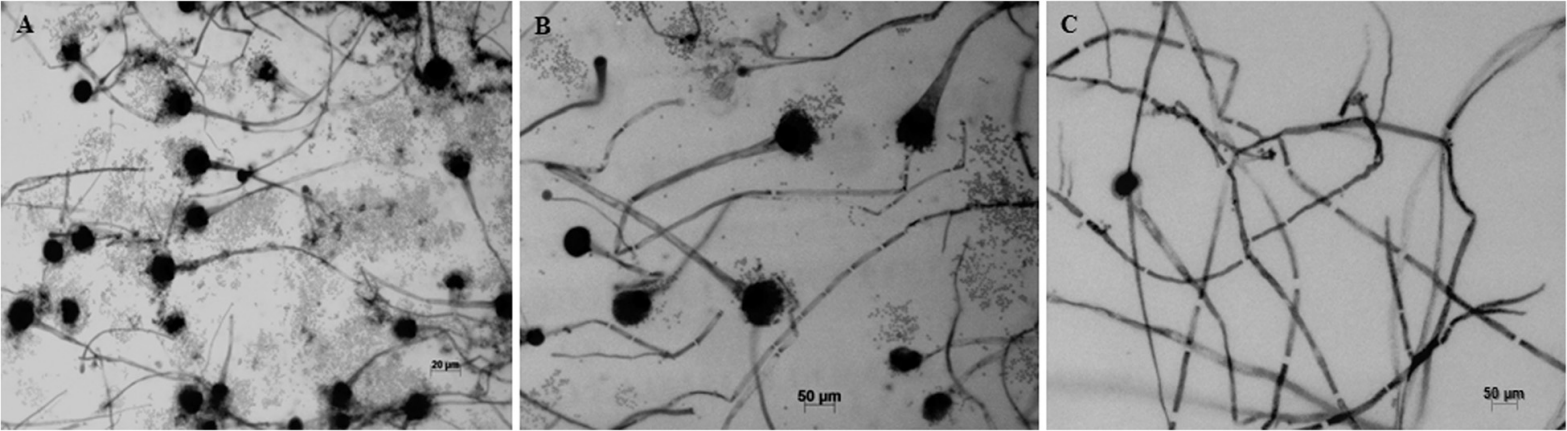
**Photomicrographs of *Aspergillus fumigatus *colonies using the cellotape flag technique preparation with lactophenol cotton blue staining.** Microscopic morphological changes in the development of conidiation of *A. fumigatus* following exposure to subinhibitory concentration of PCZ. **A**. Initial morphological aspect (control). **B**. After fifteen days. **C**. After thirty days.

There are several studies that have characterized azole resistance in *A. fumigatus,* and most recently some addressed the possible cross-resistance between environmental and medical azoles [8-11]. Our study demonstrated the time frame between the introduction of a widely used agricultural antifungal and the emergence of cross-resistance to medical triazoles. During the induction assay, we found that besides the emergence of cross-resistance, PCZ exposure caused marked morphological colony changes, both macroscopically and microscopically. Macroscopic modification of the pigmentation of *A. fumigatus* colonies, changing from the original green colour to white (Figure 1A, B and C) was remarkable at the beginning of the assay. With the increase of MIC values of PCZ the colonies became scarcer, smaller and totally white (Figure 1C). Microscopic examination showed a progressive absence of conidiation: the original strain (Figure 1A) showed normal microscopic features regarding conidiation (Figure 2A) while almost white colonies (Figure 1B) showed nearly complete absence of conidiation (Figure 2B). The totally white mycelia (Figure 1C) corresponded solely to hyphae and immature little conidiophore structures without conidia (Figure 2C). These changes in pigmentation and in conidiation as a consequence of exposure to azoles have already been reported. Varanasi and colleagues speculate that azoles may bind to a phytochrome-like regulatory molecule inhibiting the initiation and subsequent development of conidiophores in *Aspergillus* species [13]. Such mechanism of action could also explain the different levels of inhibition displayed by other tested azoles and why echinocandins and polyenes did not show this effect [13]. Notably, such morphological changes may be responsible for laboratorial diagnostic misidentification of the fungal genus/species [14]. The high MIC values for PCZ that were achieved *in vitro* maintained stable following removal of the selective pressure of the drug. For VRC, the MIC value decreased only after 30 days of incubation without the selective pressure, changing the susceptibility phenotype from resistant to intermediate. For POS, the developed MIC value also decreased but not enough to change the phenotype of resistance. Regarding ITZ, for both LMF11 and LMN60, it was observed the complete reversibility of the resistant phenotype in the absence of PCZ, ie, the MIC reverted to the initial value (susceptible). However, strain LMF05 had, since day zero, ITZ MIC of 2 mg/L, which falls in resistant category. In all the isolates conidiation reappeared together with the typical green colour of mature colonies following the removal of PCZ.

Since PCZ was responsible for the emergence of stable resistance to itself and to very important medical triazoles in *A. fumigatus*, a resistance mechanism may have been developed. Previous reports describe cyp51A mutation, efflux pump overexpression and/or target upregulation as the main mechanisms responsible for such resistance [15-17]. A clonal expansion of isolates harbouring the TR34/L98H mutation has been reported across several countries [15-18]. Interestingly, besides the fact that these resistant isolates are less genetically variable than susceptible ones, no impact on fitness was observed [18]. The phenotypic results (Figures 1 and 2) and the stability of the developed resistance (Table 1) herein reported suggest the same. Future studies aiming to assess the underlying molecular resistance mechanisms, not only from these induced resistant strains but also from isolates with naturally high MIC values to PCZ and resistant to medical azoles without previous *in vitro* induction, will certainly be our next step. Meanwhile, our study suggests that the abuse of azole antifungals in nature may cause serious human health problems since azole-resistance and cross-resistance has the potential to further compromise the efficacy of clinical azoles in the future [4,17-20]. Furthermore, we can speculate that the exposure of clinically relevant moulds other than *A. fumigatus* to agricultural azoles may also be associated with the emergence of cross-resistance to clinical azoles. Several compounds are being tested in order to find new antifungal alternatives, anticipating the possible loss of efficacy of clinical azoles [21]. On the other hand, efforts should be made to find safer compounds to use in agriculture.

## Conclusions

In order to assess the real dimension of *Aspergillus* resistance, a susceptibility test should be performed in all isolates from patients with *Aspergillus* infection. Moreover, for patients with severe infection initial combination therapy may be considered in geographical areas with high prevalence of environmental azole resistant isolates. Ultimately, surveillance studies in both clinical and in environment settings should be conducted in order to provide updated local data regarding susceptibility profiles.

## Methods

### Organisms

Two clinical isolates of *A. fumigatus,* LMF05 and LMF11, and one environmental *A. fumigatus* isolate (LMN60, recovered from a garden nearby the hospital), were used in this study. The isolates were identified as belonging to *A. fumigatus* species by macroscopic and microscopic morphology, the ability to grow at 48°C and by using MALDI-TOF MS to accurately discriminate *A. fumigatus* from a new sibling species *A. lentulus*, which cannot be distinguished by morphological characteristics or growth peculiarities [22]. Long-term preservation of conidial suspensions of the isolates was made in a GYEP medium (2% glucose, 0.3% yeast extract, 1% peptone) broth supplemented with 10% glycerol and stored at −80°C. Working cultures were subsequently maintained during 2 weeks on Sabouraud dextrose agar slants and plates at 4°C.

### Antifungal agents and susceptibility profile

PCZ is an imidazole and one of the main drugs used within European Union for crop protection [23]. This ergosterol biosynthesis inhibitor was selected as a representative of agricultural azoles after a previous MIC screening, where it showed to be the less active agricultural drug on the selected strains, ie, it had the lower MIC values, which was a prerequisite for this induction experiment. Fluconazole (FLC), VRC, POS and ITZ were selected as clinical azoles. PCZ was ressuspended in 80% acetone solution at a final concentration of 5 mg/L. Clinical azoles were dissolved in dimethysulphoxide (DMSO) to obtain stock solutions of 10 mg/L. All drugs were stored at -20°C. Broth microdilution susceptibility assay was performed according to the Clinical and Laboratory Standards Institute M38-A2 protocol in order to evaluate the initial MIC of PCZ and of all the clinical azoles [24]. Drug concentration ranged from 0.125 to 64 mg/L of FLC and PCZ; and 0.0313 to 16 mg/L of POS, VRC and ITZ. *A. fumigatus* ATCC 46645 was included for quality control of susceptibility testing. Also, FLC was used as control, since *A. fumigatus* shows a non-susceptible phenotype and MIC is most often above 64 mg/L for this species. MIC of azoles was defined as the lowest concentration of the drug that produced no visible growth following 48 hours of incubation. MIC determination was repeated at least twice.

### *In vitro* induction experiments

Induction experiments were performed with the agricultural azole PCZ. *A. fumigatus* isolates were grown on Saboraud dextrose agar at 35°C for 72 h; conidia were harvested by flooding the surface of the slants with phosphate-buffered saline (PBS) containing 0.025% (vol/vol) tween 80 while gently rocking. The conidial suspensions were then adjusted using specific spectrophotometric readings at 550 nm to a final concentration of 5×10^4^ conidia per militer [25]; one militer of each distinct isolate suspension was transferred to 9 ml of GYEP broth supplemented with sub-inhibitory concentrations of PCZ (0.06 mg/L for both LMF05 and LMF11; 0.125 mg/L for LMN60) and incubated overnight at 35°C with agitation (180 rpm). Daily, after vigorous vortexing for 60 seconds, one militer from each culture was transferred to fresh GYEP medium supplemented with PCZ and in parallel, 1 ml of culture was added with 10% glycerol and frozen at -80°C. This procedure was repeated along thirty consecutive days.

### Susceptibility testing/ Stability of *in vitro* developed resistance phenotype

MICs of PCZ were determined every ten days along the thirty days of induction assay. No official breakpoints are yet defined for PCZ; therefore, whenever a marked MIC increase was observed (four fold the initial PCZ MIC), the MIC values of clinical antifungals were determined.

In order to assess the stability of the developed MIC increment to PCZ and of the developed cross-resistance to clinical azoles, the induced strains were afterwards sub-cultured for an additional thirty days in the absence of the drug and MIC values re-determined, as previously described.

### Culture macro and micro morphology

Along the induction process, every two days, a loopful was inoculated in Saboraud Agar slants to check for viability and purity of culture. Macro and microscopical growth characteristics were registered. Colony morphology and pigmentation were recorded photographically using a Reflex Nikon D3200 Camera and images were processed by Adobe Photo Deluxe Image Processing Program. Microscopic images of hyphae changes from the original *A. fumigatus* strain and from the resistant induced strain were captured with a Zeiss-Axioplan-2 microscope equipped with Axio Cam. AxioVision 3.0 digital imaging software was used for editing.

## Competing interests

The authors declare that they have no competing interests.

## Authors’ contributions

IFR, IMM and AGR: conceived the study and designed the experiments; IFR and SF: performed the experiments; IFR, SF, JNM and PRT: analysed the data; IMM, LME, CPV and AGR: Contributed with reagents/material and analysis tools; IFR, SF, IMM and AGR: wrote and revised the manuscript. All authors read and approved the final manuscript.
